# Non-Invasive Skin Imaging Assessment of Human Stress During Head-Down Bed Rest Using a Portable Handheld Two-Photon Microscope

**DOI:** 10.3389/fphys.2022.899830

**Published:** 2022-07-25

**Authors:** Junjie Wang, Zhen Zhen, Yanqing Wang, Runlong Wu, Yanhui Hu, Qiang Fu, Yongzhi Li, Bingmu Xin, Jinping Song, Jianwei Li, Yafei Ren, Lishuang Feng, Heping Cheng, Aimin Wang, Liming Hu, Shukuan Ling, Yingxian Li

**Affiliations:** ^1^ College of Future Technology, Peking University, Beijing, China; ^2^ State Key Lab of Space Medicine Fundamentals and Application, China Astronaut Research and Training Center, Beijing, China; ^3^ Department of Environment and Life, Beijing University of Technology, Beijing, China; ^4^ Beijing Transcend Vivoscope Bio-Technology Co. Ltd., Beijing, China; ^5^ Engineering Research Center of Human Circadian Rhythm and Sleep, Space Science and Technology Institute, Shenzhen, China; ^6^ School of Instrumentation Science and Opto-Electronics Engineering, Beihang University, Beijing, China; ^7^ School of Electronics, Peking University, Beijing, China

**Keywords:** skin, portable handheld two-photon microscope, TPEF, SHG, head-down bed rest

## Abstract

Spaceflight presents a series of physiological and pathological challenges to astronauts resulting from ionizing radiation, microgravity, isolation, and other spaceflight hazards. These risks cause a series of aging-related diseases associated with increased oxidative stress and mitochondria dysfunction. The skin contains many autofluorescent substances, such as nicotinamide adenine dinucleotide phosphate (NAD(P)H), keratin, melanin, elastin, and collagen, which reflect physiological and pathological changes *in vivo*. In this study, we used a portable handheld two-photon microscope to conduct high-resolution *in vivo* skin imaging on volunteers during 15 days of head-down bed rest. The two-photon microscope, equipped with a flexible handheld scanning head, was used to measure two-photon excited fluorescence (TPEF) and second harmonic generation (SHG) images of the left forearm, left front chest, and forehead of volunteers. Changes in TPEF, SHG, and the extended SHG-to-AF(TPEF) aging index of the dermis (SAAID) were measured. It was found that TPEF intensity increased during bed rest and was restored to normal levels after recovery. Meanwhile, SHG increased slightly during bed rest, and the skin aging index increased. Moreover, we found the skin TPEF signals of the left forearm were significantly negatively associated with the oxidative stress marker malondialdehyde (MDA) and DNA damage marker 8-hydroxy-2′-desoxyguanosine (8-OHdG) values of subjects during head-down bed rest. Meanwhile, the SHG signals were also significantly negatively correlated with MDA and 8-OHDG. A significant negative correlation between the extended SAAID of the left chest and serum antioxidant superoxide dismutase (SOD) levels was also found. These results demonstrate that skin autofluorescence signals can reflect changes in human oxidant status. This study provides evidence for in-orbit monitoring of changes in human stress using a portable handheld two-photon microscope for skin imaging.

## Introduction

Spaceflight has obvious effects on the physiological systems of astronauts, including cardiovascular, musculoskeletal, respiratory, vestibular, vision, and immune systems (Demontis et al., 2017). In order to combat the adverse effects of weightlessness on astronauts and ensure their physical health and high-efficiency work capabilities during spaceflight, it is important to conduct real-time monitoring of their health and take protective measures. However, to date, no measures have been implemented to monitor their health status at a cellular level *in vivo* during spaceflight.

The skin is the largest organ of the human body and can reflect mental and physical health in different environments (Henderson et al., 2017; Li et al., 2017; Franco et al., 2022). It consists of the epidermis, dermis (including papillary dermis and reticular dermis), and subcutaneous fat. Some molecules in the skin can absorb incident photons of a specific shorter wavelength into an excited state (one-photon excitation) and quickly emit photons with a longer wavelength to return to the ground state. The emitted light is skin autofluorescence. It is known that skin endogenous fluorophores include porphyrin, advanced glycation end products (AGEs), flavin, reduced nicotinamide adenine dinucleotide (NADH), flavin adenine dinucleotide (FAD), phenylalanine, tryptophan, collagen, elastin, lipofuscin, and keratin (Richards-Kortum and Sevick-Muraca, 1996). In particular, the generation of NADH is accompanied by the formation of ATP. The measurement of NADH levels can provide relevant information on cell metabolism (Kolenc and Quinn, 2019) and oxidative stress state (Kim et al., 2017). The endogenous fluorophores of the skin can thus be used as molecular markers of pathology and physiological state.

Multiphoton microscopy is an imaging technology based on a non-linear optical process, which provides complementary modes, such as two-photon excitation fluorescence, three-photon excitation fluorescence, and harmonic generation imaging. These modes, combined with endogenous chromophores, provide opportunities for non-invasive imaging of biological tissues under natural physiological conditions. Multiphoton microscopes have been used to study aging (Koehler et al., 2006; Koehler et al., 2009; Kaatz et al., 2010; Ait El Madani et al., 2012) and cancer (Paoli et al., 2008; Dimitrow et al., 2009; Paoli et al., 2009; Pastore et al., 2017) and have been employed in cosmetic and pharmaceutical research (König et al., 2006; Ait El Madani et al., 2012). NASA/ESA launched its Skin B project, which covered skin measurements of at least five astronauts before and after spaceflight. Skin B focused on the measurement of skin physiological parameters, including the use of sub-micron high-resolution multiphoton tomography. However, this study only measured data on earth before and after the flight (Braun et al., 2019; Theek et al., 2020).

Most of the multiphoton microscope equipments are large and difficult to move, and it is hard to carry out whole body skin inspections in clinic due to their large-size probe. To meet these challenges, an attractive solution is to use a portable miniature two-photon microscope. We successfully developed a new generation high-speed and high-resolution miniature two-photon fluorescence microscope and obtained clear and stable images of brain neurons and synaptic activities in mice during free behavior (Zong et al., 2017; Zong et al., 2021). Based on similar technology, we developed a portable handheld two-photon microscope equipped with 780 nm femtosecond laser to realize a two-photon inspection of human skin *in vivo* and *in situ*.

The -6° head-down bed rest can simulate the body fluid head distribution and exercise reduction on the human body, resulting in cardiovascular dysfunction, muscle atrophy, bone loss, endocrine disorders, changes in water and salt metabolism, decreased immune function, and so on, all of which are experienced during spaceflight (Hargens and Vico, 2016). Therefore, the head-down bed rest experiment can be used to study the effect and mechanisms of, and protective measures against weightlessness on human physiological functions. This method is simple and easy to implement and is currently the most widely-used method for simulating weightlessness in the human body. However, signatures of skin autofluorescence imaging have not been characterized in individuals under head-down bed rest or mimic microgravity.

In this study, we carried out two-photon imaging to monitor human skin during 15 days of head-down bed rest and subsequent recovery using our newly-developed portable handheld two-photon microscope, which demonstrates a new approach that can non-invasively and harmlessly assess the impact of weightlessness on human stress status at the cellular level.

## Methods

### Volunteers

After nationwide recruitment for bedridden experiments through screening of gender, age, and physical and mental status, as well as two clinical physical examinations and pathological screenings, 24 volunteers were included in the experiment. The standard experiment time of the bed rest experiment was approximately 35 days, including the acclimatization period before the experiment, 15 days of bed rest, and the recovery period after bed rest. We only reported data relating to six volunteers in the bed rest group who had not been applied any special treatment.

### Ethics

All volunteers were informed of the objectives and scope of the research and provided written informed consent before the start of the research. The study protocol conformed to the ethical guidelines of the 1975 Declaration of Helsinki. The study was approved by the Ethics Committee of the Space Science and Technology Institute (Shenzhen).

### Portable Handheld Two-Photon Microscope

The portable handheld two-photon microscope consists of two main parts: a host and a probe. The host mainly consists of an 80 MHz Er-doped femtosecond fiber laser operating at 1,560 nm, a set of controller boards, and a dual channel detection module; the probe mainly consists of a miniature two-photon microscope (mTPM) head, a frequency doubling module, and a motor-driven linear stage. The host and the probe are connected via a flexible opto-electric composite cable, which contains a polarization maintaining fiber that delivers femtosecond laser pulses, a supple fiber bundle that delivers the collected signal photons, and a composite drive cable. The 1,560 fs-laser in the host, together with the frequency doubling module in the probe, work as an excitation source operating at 780 nm. The output power of the laser radiation is about 52 mW, which is considered safe for human skin imaging. The dual channel detection module is designed for autofluorescence (TPEF channel, 420–580 nm) and second harmonic generation (SHG channel, 375∼400 nm) detection with photomultiplier tube H10770PA-40. The controller boards, where a home-made control software is embedded, control the laser operation, the z-movement of the focusing optics, the 2D scanning process, the signal amplification/acquisition (16 bit), and the image generation. The mTPM head consists of a 2D MEMS scanner and home-made high NA focusing optics (NA = 0.9). The field-of-view of the optical system is 180 μm^2^ × 150 μm^2^. The working distance of the focusing optics is designed to be 1.2 mm, which takes the coverslip, optical medium, reasonable mechanical gap, and over 200 μm imaging depth into consideration. The linear stage drives the z-movement of the frequency doubling module and the mTPM head, all of which are integrated inside the handheld probe.

### Procedure

*In vivo* images of human skin were obtained from the left volar forearm, left front chest, and forehead region of healthy human subjects using the portable handheld two-photon microscope. The skin imaging at three sites was carried out 1 day before bed rest, on the third, eighth, and 15th day of bed rest, and the seventh day after recovery. The pre-imaging procedure involved wiping the skin test area with 75% alcohol cotton, placing the probe on the skin surface, finding the stratum corneum and the dermis layer according to the real-time image, and setting the parameters. Then the image scanning process starts from the skin stratum corneum to the dermis layer with step interval of 2 μm to obtain a 3D image stack of the skin. Volunteers underwent forearm venipuncture in the morning at prescribed time points (1 day before bed rest, and at 7 and 15 days during bed rest). Collected blood samples were stored in a freezer at −80°C for subsequent testing. Serum was tested for oxidative stress indicators, including superoxide dismutase (SOD), malondialdehyde (MDA), and 8-hydroxydeoxyguanosine (8-OHDG).

### Image Analysis

Image registration was first performed. Since the basale cell layer is located on the papillary dermis and is uneven, for the images obtained with the TPEF channel, images from the first layer of basale cells that appeared to the previous layer of collagen fibers that appeared were selected as the whole basale cell layer for processing. The cell layer was processed and Fiji (ImageJ 1.52i) was used to subtract the background of the selected image. The maximum value projection was then performed on the basale cell layer stack composed of all the images of the selected layers to obtain one image. We selected the region where the basale cells are concentrated as ROI and then calculated the average gray-scale value. For the images obtained with the SHG channel, the images of the same ROI and same timepoint performed the same operation as the TPEF channel images. We selected the same number of layers, subtracted the background, undertook maximum value projection, selected the same ROI, and calculated the mean gray value. Finally, we extended the concept of SAAID from dermis to epidermis using the same equation, SAAID = (SHG − TPEF)/(SHG + TPEF). Here, the SAAID can probably be considered a normalization of TPEF signals in the innermost epidermis versus SHG.

### Statistical Analysis

Statistics from all volunteers by time point and GraphPad Prism 9.0.0 were used for statistical analyses and graphing. Statistical differences among groups were analyzed by one-way repeated measures ANOVA, followed by Tukey’s test to determine group differences in the study parameters. Pearson correlation coefficient (*R* ^2^) was performed to assess the correlation between two variables. Comparisons with *p* < 0.05 were considered to be statistically significant.

## Results

### Skin Imaging Using a Portable Handheld Two-Photon Microscope

**FIGURE 1:**
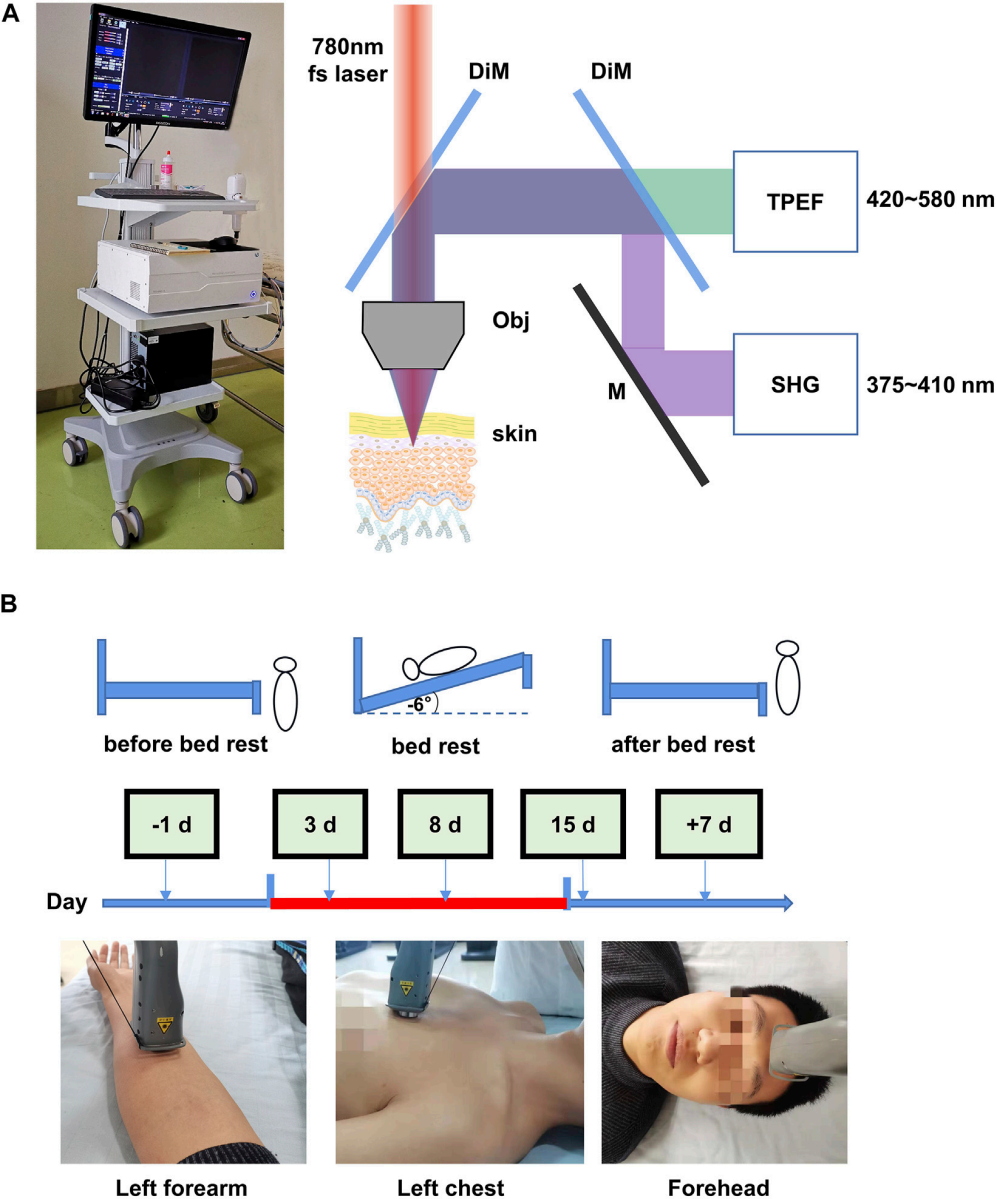
Optical biopsy of *in situ* human skin during 15 days of head-down bed rest. **(A)** Corresponding two-photon excited fluorescence (TPEF) and second harmonic generation (SHG) fitted images of the skin obtained from different depths below the skin surface, as shown in the images. **(B)** Measurement schedule. The indicated measurements were performed at each time point.

**FIGURE 2:**
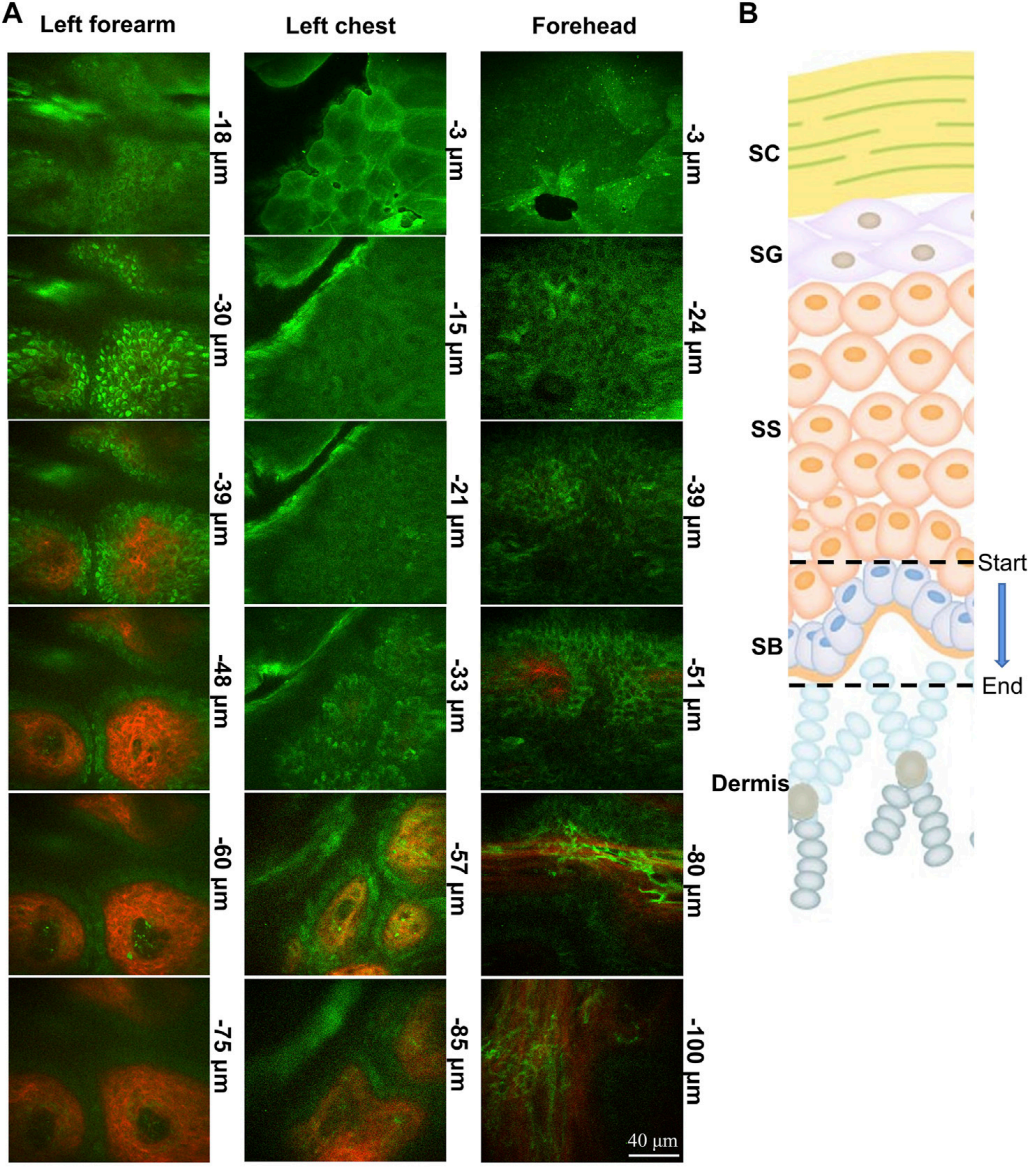
Portable handheld two-photon microscope imaging of a skin structure. **(A,B)** Image of the clinical device portable two-photon microscope to perform non-invasive, label-free, rapid *in vivo* histology. Photograph of indicated parts of the body coupled to the probe of portable two-photon microscope imaging system during imaging. TPEF (green) and SHG (red) images of skin obtained from different depths below the skin surface. SC, stratum corneum; SG, stratum granulosum; SS, stratum spinosum; SB, stratum basale.

The skin contains numerous endogenous fluorophores. The two-photon microscope can obtain label-free *in vivo* images of skin noninvasively with sub-cellular resolution from the skin surface down to a depth of over 120 μm. In this study, a portable handheld two-photon microscope was used. This microscope is easy to carry and can flexibly detect the skin of different parts of the human body (Figures 1A,B). The two-photon microscope provides clear images of skin structure by acquiring two-photon excited fluorescence (TPEF) and second harmonic generation (SHG) images simultaneously. The stratum corneum, stratum granulosum, stratum spinosum and stratum basale of the skin can be seen from top to bottom (Figures 2A,B). The TPEF signal at 420–580 nm from the spinous layer and basale cell layer is mainly NADH, while the SHG signal depicts the collagen network. Based on the TPEF and SHG data, SAAID was calculated at different time points before, during, and after bed rest.

### Changes in the Two-Photon Excited Fluorescence Index in the Basale Cell Layer of the Epidermis at Different Time Points

**FIGURE 3:**
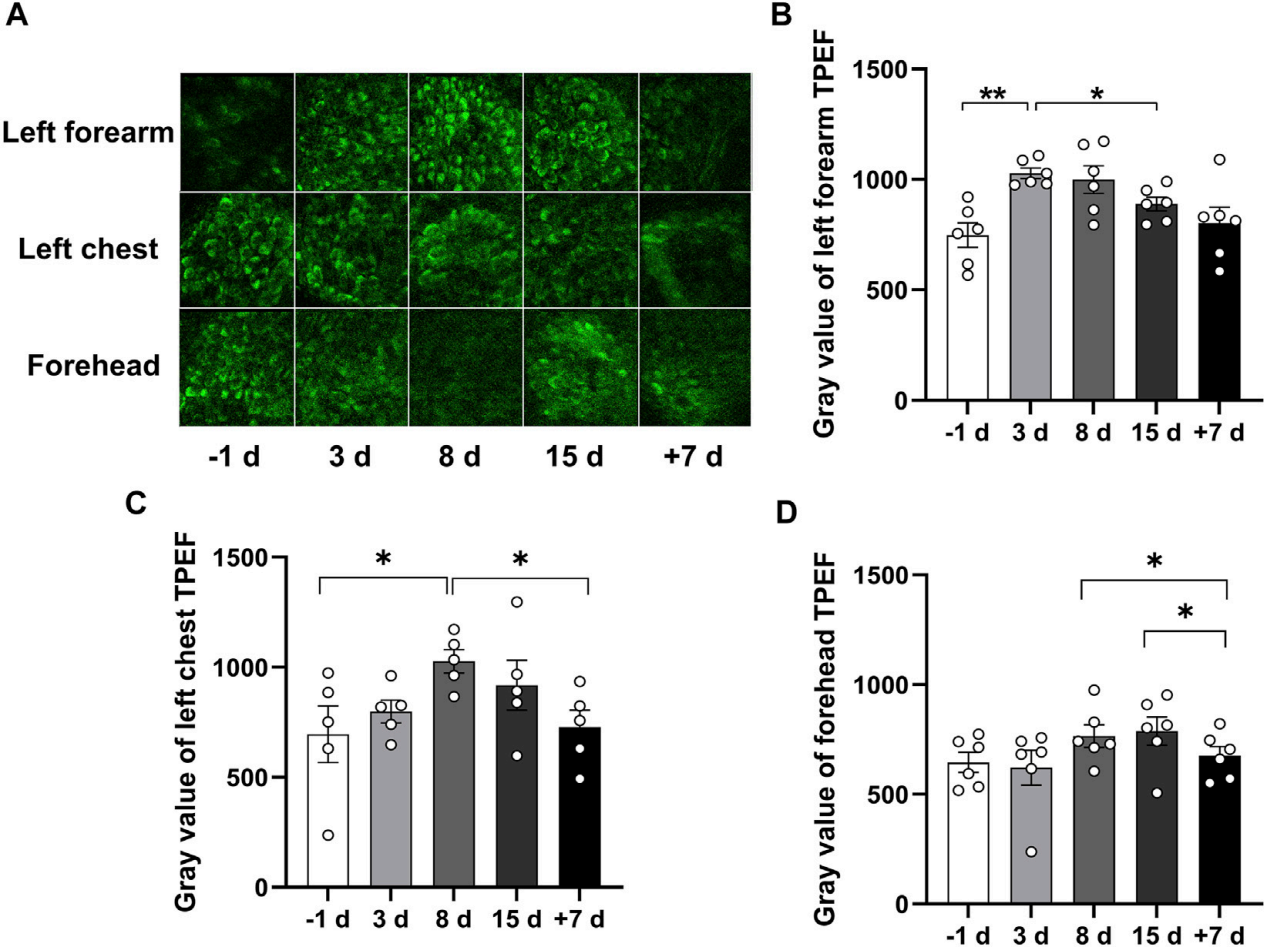
Qualitative comparison of skin TPEF during 15 days of head-down bed rest. **(A)** Typical images of TPEF intensity in the subcutaneous basale cell layer in different parts of the skin at each time point. **(B–D)** Changes of TPEF intensity in the skin basale cell layer of left forearm, left chest and forehead during 15 days of head-down bed rest. Data represent mean ± SEM. Significant values are determined as *p < 0.05, **p < 0.01 (Tukey’s multiple comparisons paired test).

TPEF can be used to provide high-resolution skin morphology and yield a complete picture of the skin’s endogenous fluorophores. Stratum basal, which is the innermost layer of the epidermis, can continually divide and be pushed towards the skin surface. The stratum basale layer cells are sensitive to vascular oxygen, whereas the cells of the upper layers, such as stratum granulosum, are mostly supplied by atmospheric oxygen (Balu et al., 2013). To investigate the effect of 15 days of head-down bed rest on the NADH of basale cells, we examined the stratum basale TPEF of the left forearm, left front chest, and forehead skin at different time points. As shown in Figure 3A, the image of TPEF displayed the structure of basale cells. The basale cell TPEF intensity was analyzed. The results showed that the TPEF of the left forearm increased at the third day of bed rest compared with the period prior to bed rest, while the TPEF of the left front chest and forehead increased on the eighth day of bed rest. The TPEF showed a decreasing trend thereafter but did not return to the original level at 15 days of bed rest. However, the TPEF can be restored to normal levels after 7 days of recovery (Figures 3B,C). These results indicate that 15 days of head-down bed rest led to the alteration of NADH in basale cells.

### Quantitative Comparison of Second Harmonic Generation Signal at Different Time Points

**FIGURE 4:**
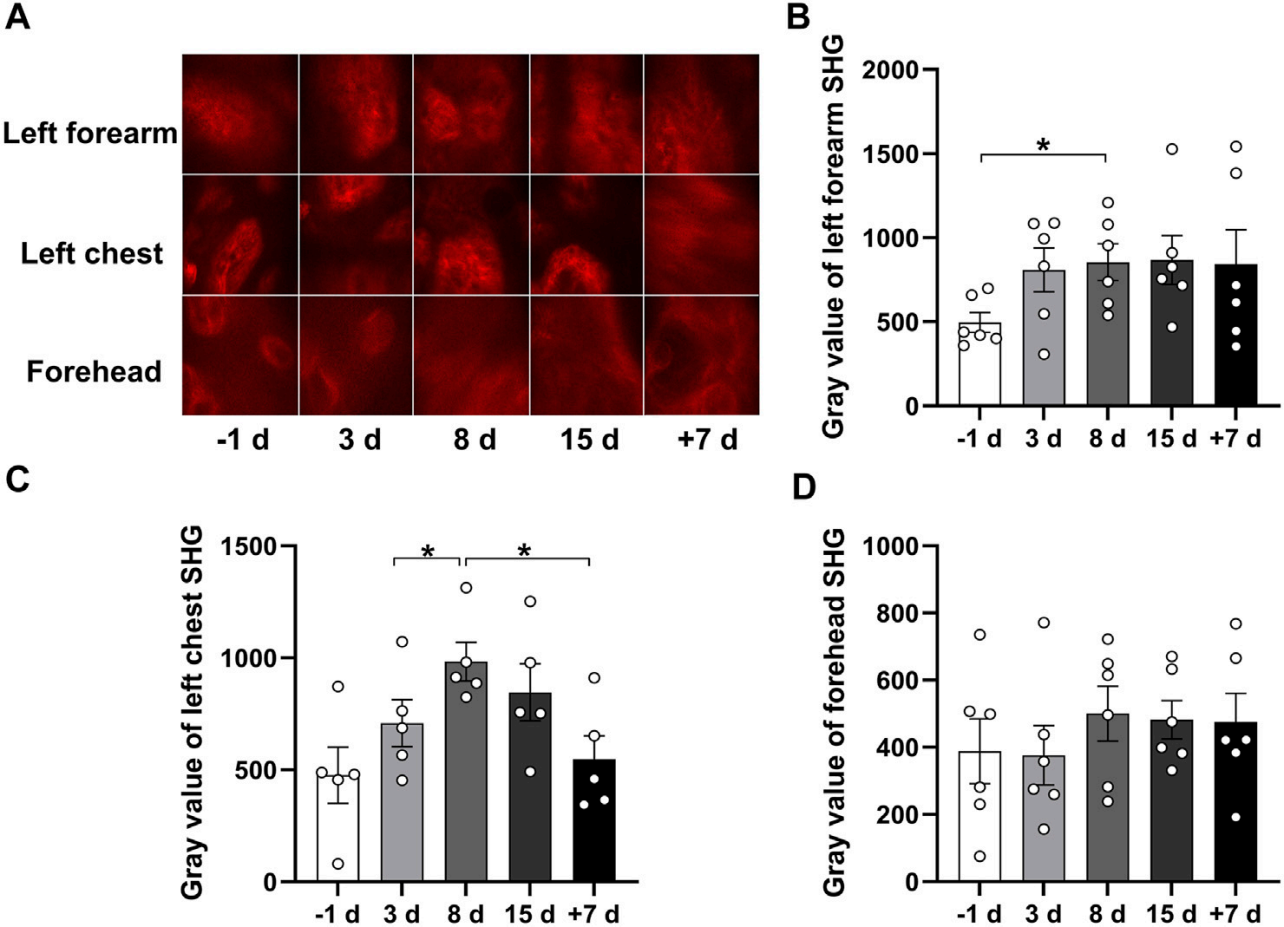
Qualitative comparison of skin SHG during 15 days of head-down bed rest. **(A)** Typical images of SHG intensity in the subcutaneous basals cell layer in different parts of the skin as a function of time in bed. **(B–D)** Changes of SHG intensity in the skin basale cell layer of left forearm, left chest, and forehead during 15 days of head-down bed rest. Data represent mean ± SEM. Significant values are determined as *p < 0.05 (Tukey’s multiple comparisons paired test).

Skin structural proteins can produce the SHG signal, and the strongest signal is induced by collagen. To investigate the effect of 15 days of head-down bed rest on skin structural proteins *in vivo*, we examined the SHG signal of the left forearm, left front chest, and forehead skin at different time points. In Figure 4A, images of SHG in the epidermis are shown. Analysis of the SHG signal revealed an increase on day 8 of bed rest compared with the period before bed rest, which was then restored to the normal level after 7 days of recovery (Figures 4B,C). These results indicate that 15 days of head-down bed rest induced the change in skin structural proteins.

### Changes in SHG-to-AF(TPEF) Aging Index of Dermis at Different Time Points

**FIGURE 5:**
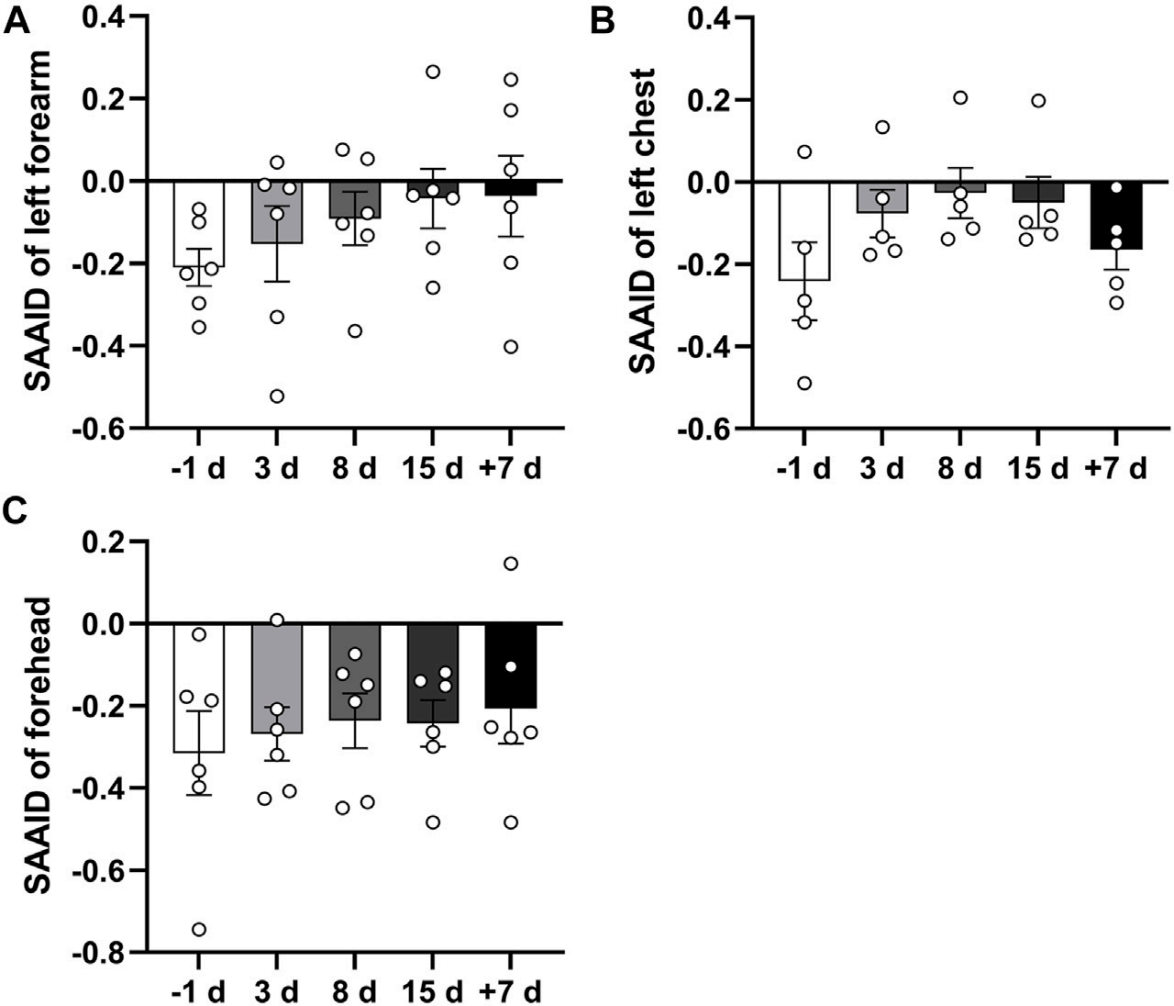
Qualitative comparison of skin SAAID during 15 days of head-down bed rest. **(A–C)** Changes in SAAID index calculated from TPEF and SHG values of left forearm, left anterior chest, and forehead during 15-day bed rest with head down. Data represent mean ± SEM. Significant values are determined as *p < 0.05 (Tukey’s multiple comparisons paired test).

The SAAID here can be considered a normalization of TPEF signals in the innermost epidermis versus SHG. We analyzed the SAAID combined with TPEF and SHG signals of the left forearm, left front chest, and forehead skin at different time points. The results showed that the SAAID has an increasing trend after 15 days of head-down bed rest (Figures 5A–C). These results suggest that 15 days of head-down bed rest might have an impact on skin aging.

### Correlation of Serum Redox Status Marker Levels With Two-Photon Excited Fluorescence and SHG-to-AF (TPEF) Aging Index of Dermis

**FIGURE 6:**
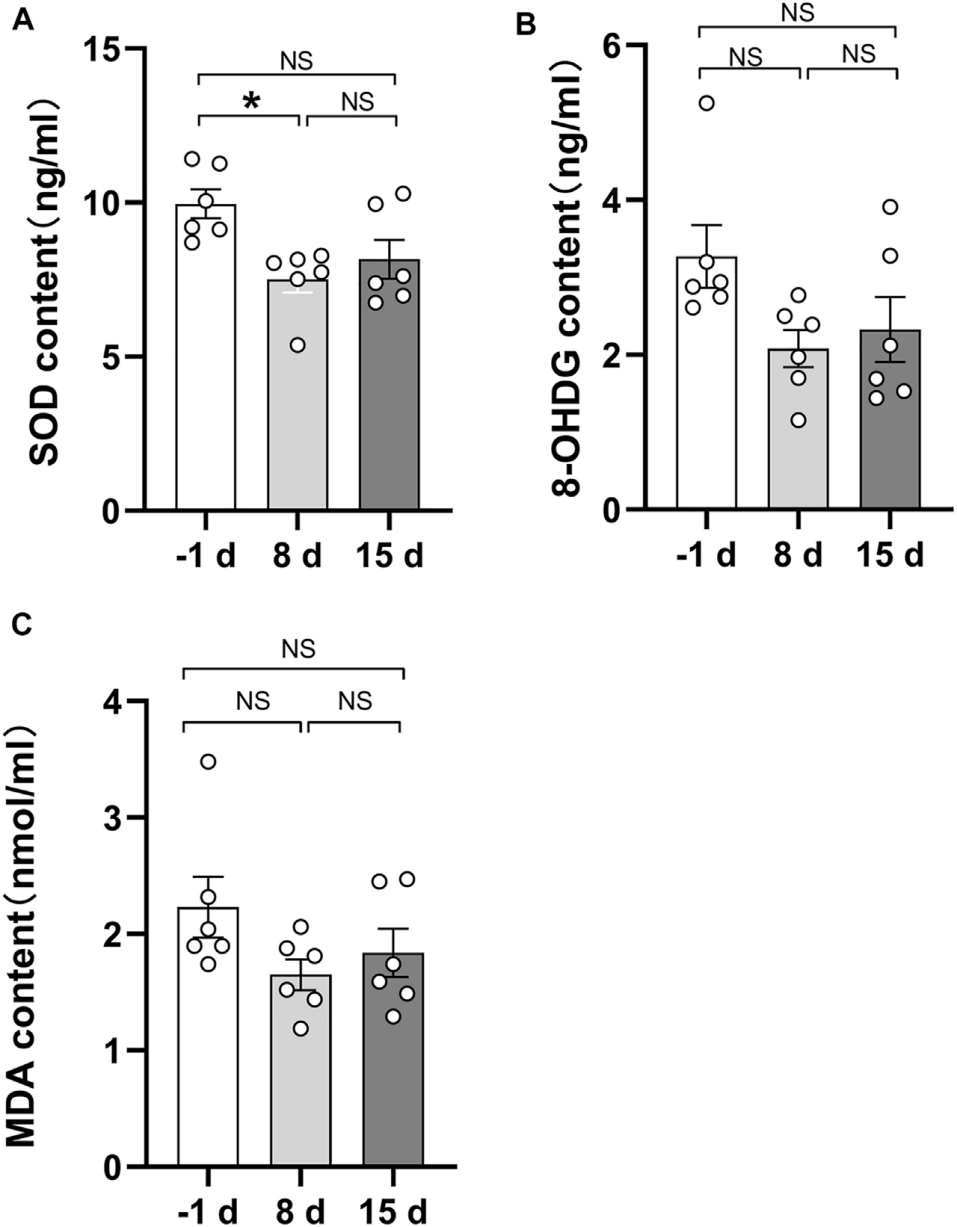
Quantitative comparison of serum biochemical indicators during 15 days of head-down bed rest. **(A)** Changes of serum superoxide dismutase content during 15 days of head-down bed rest. Data represent mean ± SEM. Significant values are determined as *p < 0.05 (Tukey’s multiple comparisons paired test). **(B)** Changes of serum 8-hydroxydeoxyguanosine during 15 days of head-down bed rest. Data represent mean ± SEM. (Tukey’s multiple comparisons paired test). **(C)** Changes of serum malondialdehyde content during 15 days of head-down bed rest. Data represent mean ± SEM. (Tukey’s multiple comparisons paired test).

**FIGURE 7:**
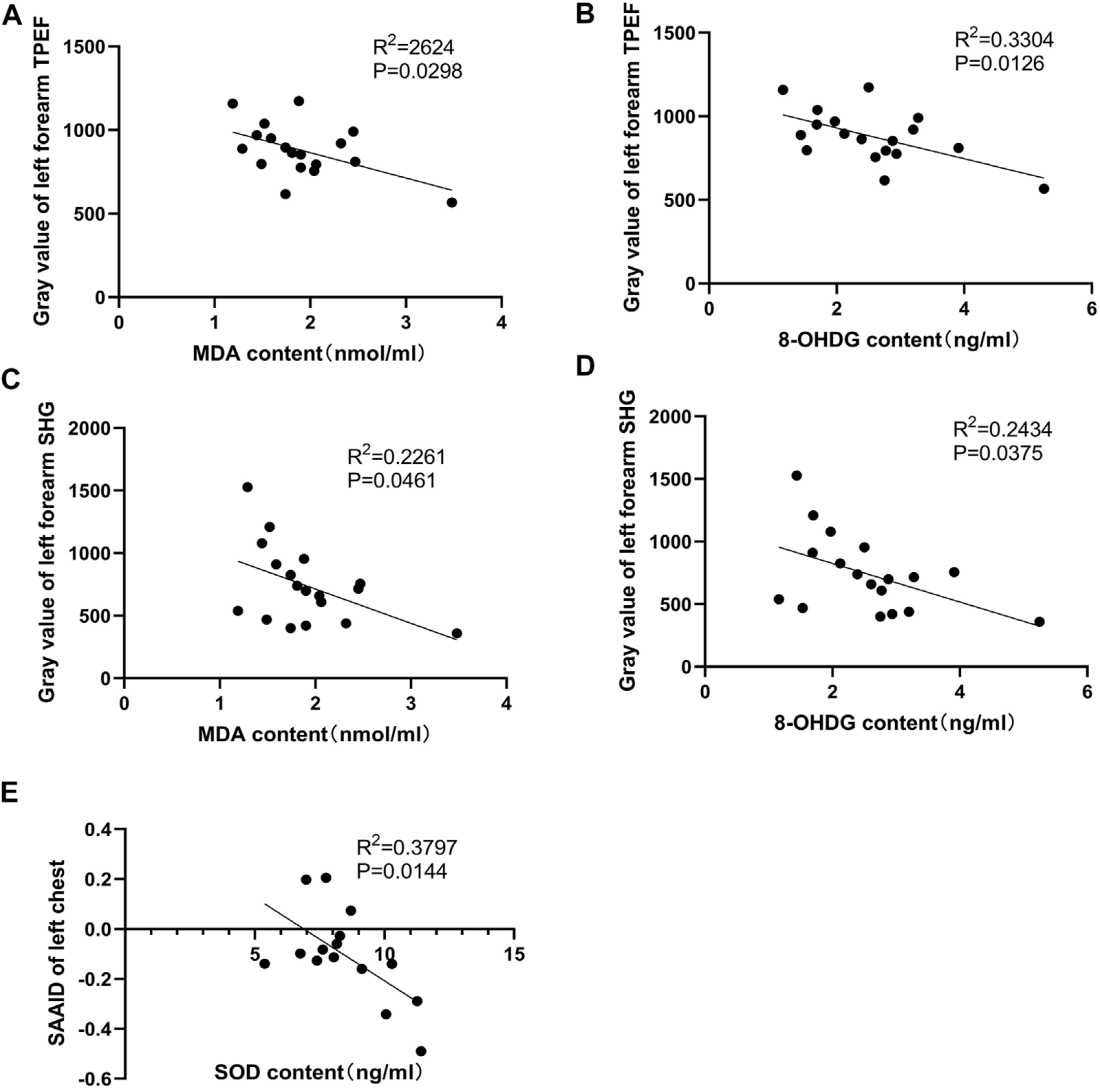
Pearson correlation coefficients of two-photon detection indexes and serum biochemical indicators. **(A,B)** Pearson correlation coefficients of serum MDA, 8-OHDG and gray value of left forearm skin TPEF. There was a significant negative correlation. **(C,D)** Pearson correlation coefficients of serum MDA, 8-OHDG, and gray value of left forearm skin SHG. The left forearm SHG gray value was significantly negatively correlated with serum MDA and 8-OHDG levels. **(E)** The SAAID index calculated by TPEF and SHG intensity of left anterior chest were significantly negatively correlated with serum SOD.

As indicated by previous research, head-down bed rest is a useful and reliable simulation model for physiological changes during spaceflight (Jost, 2008). Blood samples were collected during 15 days of head-down bed rest. Serum levels of oxidative markers (MDA, 8-OHDG, and SOD) were measured. The results showed that the serum SOD value after 8 days of head-down bed rest was significantly lower compared with the period before bed rest (Figure 6A), By contrast, the levels of MDA and 8-OHDG remained unchanged (Figures 6B,C). To determine the correlation of serum redox status marker levels with the value of TPEF and SAAID, we used Pearson’s method to analyze the multiple correlation coefficient. As shown in Figures 7A,B, the TPEF of the left forearm was significantly negatively associated with the serum MDA and 8-OHDG value of subjects at total time points. Meanwhile, the SHG of the left forearm was also significantly negatively associated with the serum MDA and 8-OHDG value of subjects (Figures 7C,D). The results showed a significant negative correlation between the SAAID of the left chest and SOD (Figure 7E). All these results suggest that measurement of the skin TPEF, SHG, and SAAID by a portable handheld two-photon microscope *in vivo* can be used to assess the oxidative and antioxidant capacity of the body during spaceflight.

## Discussion

This study is the first to report on the noninvasive assessment of human stress status during head-down bed rest using a portable handheld two-photon microscope. The signals of fluorescent substances and the second harmonic generation signal of structural proteins around the dermis-epidermis junction were used to observe the dynamic changes of the basale layer cells. The results showed that 15 days of head-down bed rest had induced the changes in skin TPEF, SHG, and SAAID. Importantly, we found the skin TPEF signals of the left forearm were significantly negatively associated with the serum oxidative markers -MDA and the 8-OHDG value of subjects during head-down bed rest. Moreover, the SHG signals also had a significant negative correlation with MDA and 8-OHDG, and a significant negative correlation between SAAID of the left chest with serum SOD values was also found. This study demonstrates that the portable handheld two-photon microscope for skin imaging can be used to monitor human stress during spaceflight.

Two-photon microscopy has different advantages and characteristics for cell imaging, tissue imaging, and *in vivo* imaging studies. Some studies have applied it to observing the morphological characteristics of different lesions, such as basal cell carcinoma and malignant melanoma (Balu et al., 2014; Kiss et al., 2019). For *in vivo* imaging research, two-photon microscopy systems usually use infrared laser as the excitation light source. This affords good penetration of biological tissues and can reach an imaging depth of hundreds of microns and can thus achieve dynamic, real-time, and continuous monitoring of molecular events *in vivo*. Since the two-photon microscopy imaging technology based on SHG and TPEF was first used in biomedicine, it has received extensive attention for its unique advantages. This technology is mainly used in fibrosis diagnosis and the grading of tissues and organs (Sun et al., 2008), discrimination of tumors (van Huizen et al., 2020), and morphological observation of the living tissue cell matrix *in vivo* or *in vitro* (Zoumi et al., 2002)*.* The endogenous fluorophores of the skin allow label-free visualization of the cellular and subcellular features of various skin layers (Yew et al., 2014). In this study, we used a portable handheld two-photon microscope for skin imaging during head-down bed rest. It was found that TPEF intensity increased during bed rest and was restored to the normal level after recovery. Meanwhile, SHG increased slightly during bed rest, and the SAAID also increased. These findings indicate that head down bed rest induced changes to the skin’s physiological parameters.

Free radicals can cause lipid peroxidation to form peroxides, among which 8-hydroxydeoxyguanosine (8-OHDG) and malondialdehyde (MDA) are markers of DNA and lipid oxidation. These two serum indexes can directly reflect the effect of oxidative stress. SOD is one of the important components of the body’s antioxidant system. It can remove harmful substances such as free radicals generated by oxidative stress and reflect the body’s antioxidative function (Demirci-Çekiç et al., 2022). Spaceflight can cause the imbalance of redox homeostasis in human and animal blood, urine, and tissue (Goodwin and Christofidou-Solomidou, 2018) In this study, the serum SOD value after 8 days of head-down bed rest had significantly decreased compared with the period before head-down bed rest. By contrast, the levels of MDA and 8-OHDG remained unchanged. This is the same as previous data from male cohorts indicating significant inactivity-provoked antioxidant status reduction (Powers et al., 2007) in contrast to the data from female cohorts (Debevec et al., 2016). Whereas previous studies showed bed rest exposure induced the increase of blood oxidative stress markers (Margaritis et al., 2009; Debevec et al., 2016), the decrease in serum SOD and the lack of increase in MDA and 8-OHDG in our study can be attributed to lower basale stress with the short-term bed rest. In this study, we analyzed these three serum indicators during 15 days of head-down bed rest and performed correlation analysis with the TPEF, SHG, and SAAID of the skin. We found the skin’s TPEF and SHG signals were significantly negatively associated with serum MDA and 8-OHDG during head-down bed rest. Moreover, a significant negative correlation between the SAAID of the left chest and serum SOD values was found. These results demonstrate the skin’s two-photon fluorescence microscope signals can reflect changes in human oxidative status.

An important metabolic fluorophore in the epidermis is NADH. The two-photon microscope can observe the fluorescence of NADH, which acts as an electron carrier from the Krebs cycle to the respiratory chain (Mayevsky & Rogatsky, 2007; Balu et al., 2013). Mellem et al. (2017)used MitoTracker Red CMXRos to locate mitochondria and compare them with the autofluorescence of NADH observed by a confocal scanning microscope. They found that the morphology of stained mitochondria was almost identical to the autofluorescence signal of NADH (Mellem et al., 2017). NADH mainly exists in mitochondria. The NADH level in basale layer cells is sensitive to vascular oxygen supply. Moreover, the changes in NADH fluorescence of human epidermal cells under conditions of blood-supplied oxygen deprivation can reflect mitochondrial function and cellular metabolism. This study suggests the potential application of the portable handheld two-photon microscope in monitoring mitochondrial function.

The limitation of this study is that the basale layer contains melanin that can also emit fluorescence in the skin under these imaging conditions. Fortunately, the skin type of volunteers is close to Fitzpatrick II∼III, so the influence of melanin is not obvious. Furthermore, the volunteers had no chance of exposure to sunlight in the head-down bed rest experiment, so the melanin would have decreased gradually or remained at a constant low level, which means that the fluorescence contribution of melanin would have had a decreasing trend or at least no impact on the change in fluorescence. Therefore, we speculate that the increase of the TPEF during bed rest was mainly due to the change in NADH.

In summary, this study carried out a non-invasive assessment of human stress status during 15 days of head-down bed rest and subsequent recovery period using a portable handheld two-photon microscope, providing us with a new method that can non-invasively and harmlessly assess the impact of weightlessness on human stress status at the cellular level. Our results add a new dimension to the study of the physiological changes during head down bed rest. In the future, the portable handheld two-photon microscope can be used as a powerful tool to monitor astronauts' health in spaceflight.

## Data Availability

The original contributions presented in the study are included in the article/supplementary material, and further inquiries can be directed to the corresponding authors.

## References

[B1] Ait El Madani H. Tancrède-Bohin E. Bensussan A. Colonna A. Dupuy A. Bagot M. (2012). *In Vivo* multiphoton Imaging of Human Skin: Assessment of Topical Corticosteroid-Induced Epidermis Atrophy and Depigmentation. J. Biomed. Opt. 17, 026009. 10.1117/1.jbo.17.2.026009 22463041

[B2] Balu M. Kelly K. M. Zachary C. B. Harris R. M. Krasieva T. B. König K. (2014). Distinguishing between Benign and Malignant Melanocytic Nevi by *In Vivo* Multiphoton Microscopy. Cancer Res. 74, 2688–2697. 10.1158/0008-5472.can-13-2582 24686168 PMC4024350

[B3] Balu M. Mazhar A. Hayakawa C. K. Mittal R. Krasieva T. B. König K. (2013). *In Vivo* multiphoton NADH Fluorescence Reveals Depth-dependent Keratinocyte Metabolism in Human Skin. Biophysical J. 104, 258–267. 10.1016/j.bpj.2012.11.3809 PMC3540245 23332078

[B4] Braun N. Binder S. Grosch H. Theek C. Ülker J. Tronnier H. (2019). Current Data on Effects of Long-Term Missions on the International Space Station on Skin Physiological Parameters. Skin. Pharmacol. Physiol. 32, 43–51. 10.1159/000494688 30485843

[B5] Debevec T. Pialoux V. Ehrström S. Ribon A. Eiken O. Mekjavic I. B. (2016). FemHab: The Effects of Bed Rest and Hypoxia on Oxidative Stress in Healthy Women. J. Appl. Physiol. (1985) 120, 930–938. 10.1152/japplphysiol.00919.2015 26796757

[B6] Demirci-Çekiç S. Özkan G. Avan A. N. Uzunboy S. Çapanoğlu E. Apak R. (2022). Biomarkers of Oxidative Stress and Antioxidant Defense. J. Pharm. Biomed. analysis 209, 114477. 10.1016/j.jpba.2021.114477 34920302

[B7] Demontis G. C. Germani M. M. Caiani E. G. Barravecchia I. Passino C. Angeloni D. (2017). Human Pathophysiological Adaptations to the Space Environment. Front. Physiol. 8, 547. 10.3389/fphys.2017.00547 28824446 PMC5539130

[B8] Dimitrow E. Ziemer M. Koehler M. J. Norgauer J. König K. Elsner P. (2009). Sensitivity and Specificity of Multiphoton Laser Tomography for *In Vivo* and *Ex Vivo* Diagnosis of Malignant Melanoma. J. Investigative Dermatology 129, 1752–1758. 10.1038/jid.2008.439 19177136

[B9] Franco A. C. Aveleira C. Cavadas C. (2022). Skin Senescence: Mechanisms and Impact on Whole-Body Aging. Trends Mol. Med. 28, 97–109. 10.1016/j.molmed.2021.12.003 35012887

[B10] Goodwin T. J. Christofidou-Solomidou M. (2018). Oxidative Stress and Space Biology: An Organ-Based Approach. Int. J. Mol. Sci. 19, 959. 10.3390/ijms19040959 PMC5979446 29570635

[B11] Hargens A. R. Vico L. (2016). Long-duration Bed Rest as an Analog to Microgravity. J. Appl. Physiol. (Bethesda, Md 1985) 120, 891–903. 10.1152/japplphysiol.00935.2015 26893033

[B12] Henderson A. J. Lasselin J. Lekander M. Olsson M. J. Powis S. J. Axelsson J. (2017). Skin Colour Changes during Experimentally-Induced Sickness. Brain, Behav. Immun. 60, 312–318. 10.1016/j.bbi.2016.11.008 27847284

[B13] Jost P. D. (2008). Simulating Human Space Physiology with Bed Rest. Hippokratia 12 (Suppl. 1), 37–40. PMC2577398 19048091

[B14] Kaatz M. Sturm A. Elsner P. König K. Bückle R. Koehler M. J. (2010). Depth-resolved Measurement of the Dermal Matrix Composition by Multiphoton Laser Tomography. Skin Res. Technol. official J. Int. Soc. Bioeng. Skin (ISBS) Int. Soc. Digital Imaging Skin (ISDIS) Int. Soc. Skin Imaging (ISSI) 16, 131–136. 10.1111/j.1600-0846.2009.00423.x 20456091

[B15] Kim S.-Y. Cohen B. M. Chen X. Lukas S. E. Shinn A. K. Yuksel A. C. (2017). Redox Dysregulation in Schizophrenia Revealed by *In Vivo* NAD+/NADH Measurement. Schbul 43, 197–204. 10.1093/schbul/sbw129 PMC5216857 27665001

[B16] Kiss N. Haluszka D. Lőrincz K. Gyöngyösi N. Bozsányi S. Bánvölgyi A. (2019). Quantitative Analysis on *Ex Vivo* Nonlinear Microscopy Images of Basal Cell Carcinoma Samples in Comparison to Healthy Skin. Pathol. Oncol. Res. 25, 1015–1021. 10.1007/s12253-018-0445-1 29981012

[B17] Koehler M. J. König K. Elsner P. Bückle R. Kaatz M. (2006). *In Vivo* assessment of Human Skin Aging by Multiphoton Laser Scanning Tomography. Opt. Lett. 31, 2879–2881. 10.1364/ol.31.002879 16969409

[B18] Koehler M. J. Preller A. Kindler N. Elsner P. König K. Bückle R. (2009). Intrinsic, Solar and Sunbed-Induced Skin Aging Measuredin Vivoby Multiphoton Laser Tomography and Biophysical Methods. Skin Res. Technol. official J. Int. Soc. Bioeng. Skin (ISBS) Int. Soc. Digital Imaging Skin (ISDIS) Int. Soc. Skin Imaging (ISSI) 15, 357–363. 10.1111/j.1600-0846.2009.00372.x 19624433

[B19] Kolenc O. I. Quinn K. P. (2019). Evaluating Cell Metabolism through Autofluorescence Imaging of NAD(P)H and FAD. Antioxidants redox Signal. 30, 875–889. 10.1089/ars.2017.7451 PMC6352511 29268621

[B20] König K. Ehlers A. Stracke F. Riemann I. (2006). *In Vivo* drug Screening in Human Skin Using Femtosecond Laser Multiphoton Tomography. Skin. Pharmacol. Physiol. 19, 78–88. 10.1159/000091974 16685146

[B21] Li A. W. Yin E. S. Stahl M. Kim T. K. Panse G. Zeidan A. M. (2017). The Skin as a Window to the Blood: Cutaneous Manifestations of Myeloid Malignancies. Blood Rev. 31, 370–388. 10.1016/j.blre.2017.07.003 28732587

[B22] Margaritis I. Rousseau A. S. Marini J. F. Chopard A. (2009). Does Antioxidant System Adaptive Response Alleviate Related Oxidative Damage with Long Term Bed Rest? Clin. Biochem. 42, 371–379. 10.1016/j.clinbiochem.2008.10.026 19059391

[B23] Mayevsky A. Rogatsky G. G. (2007). Mitochondrial Function *In Vivo* Evaluated by NADH Fluorescence: from Animal Models to Human Studies. Am. J. Physiology-Cell Physiology 292, C615–C640. 10.1152/ajpcell.00249.2006 16943239

[B24] Mellem D. Sattler M. Pagel-Wolff S. Jaspers S. Wenck H. Rübhausen M. A. (2017). Fragmentation of the Mitochondrial Network in Skin *In Vivo* . PloS one 12, e0174469. 10.1371/journal.pone.0174469 28644888 PMC5482427

[B25] Paoli J. Smedh M. Ericson M. B. (2009). Multiphoton Laser Scanning Microscopy-A Novel Diagnostic Method for Superficial Skin Cancers. Seminars Cutan. Med. Surg. 28, 190–195. 10.1016/j.sder.2009.06.007 19782943

[B26] Paoli J. Smedh M. Wennberg A.-M. Ericson M. B. (2008). Multiphoton Laser Scanning Microscopy on Non-melanoma Skin Cancer: Morphologic Features for Future Non-invasive Diagnostics. J. Investigative Dermatology 128, 1248–1255. 10.1038/sj.jid.5701139 17989735

[B27] Pastore M. N. Studier H. Bonder C. S. Roberts M. S. (2017). Non-invasive Metabolic Imaging of Melanoma Progression. Exp. Dermatol 26, 607–614. 10.1111/exd.13274 27992081

[B28] Powers S. K. Kavazis A. N. McClung J. M. (2007). Oxidative Stress and Disuse Muscle Atrophy. J. Appl. Physiol. (1985) 102, 2389–2397. 10.1152/japplphysiol.01202.2006 17289908

[B29] Richards-Kortum R. Sevick-Muraca E. (1996). Quantitative Optical Spectroscopy for Tissue Diagnosis. Annu. Rev. Phys. Chem. 47, 555–606. 10.1146/annurev.physchem.47.1.555 8930102

[B30] Sun W. Chang S. Tai D. C. S. Tan N. Xiao G. Tang H. (2008). Nonlinear Optical Microscopy: Use of Second Harmonic Generation and Two-Photon Microscopy for Automated Quantitative Liver Fibrosis Studies. J. Biomed. Opt. 13, 064010. 10.1117/1.3041159 19123657

[B31] Theek C. Tronnier H. Heinrich U. Braun N. (2020). Surface Evaluation of Living Skin (SELS) Parameter Correlation Analysis Using Data Taken from Astronauts Working under Extreme Conditions of Microgravity. Skin. Res. Technol. 26, 105–111. 10.1111/srt.12771 31541489

[B32] van Huizen L. M. G. Radonic T. van Mourik F. Seinstra D. Dickhoff C. Daniels J. M. A. (2020). Compact Portable Multiphoton Microscopy Reveals Histopathological Hallmarks of Unprocessed Lung Tumor Tissue in Real Time. Transl. Biophot. 2, e202000009. 10.1002/tbio.202000009 PMC8311669 34341777

[B33] Yew E. Rowlands C. So P. T. C. (2014). Application of Multiphoton Microscopy in Dermatological Studies: a Mini-Review. J. Innov. Opt. Health Sci. 07, 1330010. 10.1142/s1793545813300103 PMC4112132 25075226

[B34] Zong W. Wu R. Chen S. Wu J. Wang H. Zhao Z. (2021). Miniature Two-Photon Microscopy for Enlarged Field-Of-View, Multi-Plane and Long-Term Brain Imaging. Nat. Methods 18, 46–49. 10.1038/s41592-020-01024-z 33408404

[B35] Zong W. Wu R. Li M. Hu Y. Li Y. Li J. (2017). Fast High-Resolution Miniature Two-Photon Microscopy for Brain Imaging in Freely Behaving Mice. Nat. Methods 14, 713–719. 10.1038/nmeth.4305 28553965

[B36] Zoumi A. Yeh A. Tromberg B. J. (2002). Imaging Cells and Extracellular Matrix *In Vivo* by Using Second-Harmonic Generation and Two-Photon Excited Fluorescence. Proc. Natl. Acad. Sci. U.S.A. 99, 11014–11019. 10.1073/pnas.172368799 12177437 PMC123202

